# MeDor: a metaserver for predicting protein disorder

**DOI:** 10.1186/1471-2164-9-S2-S25

**Published:** 2008-09-16

**Authors:** Philippe Lieutaud, Bruno Canard, Sonia Longhi

**Affiliations:** 1 Architecture et Fonction des Macromolécules Biologiques, UMR 6098 CNRS et Universités Aix-Marseille I et II, 163 Avenue de Luminy, Case 932, 13288 Marseille Cedex 09, France

## Abstract

**Background:**

We have previously shown that using multiple prediction methods improves the accuracy of disorder predictions. It is, however, a time-consuming procedure, since individual outputs of multiple predictions have to be retrieved, compared to each other and a comprehensive view of the results can only be obtained through a manual, fastidious, non-automated procedure. We herein describe a new web metaserver, MeDor, which allows fast, simultaneous analysis of a query sequence by multiple predictors and provides a graphical interface with a unified view of the outputs.

**Results:**

MeDor was developed in Java and is freely available and downloadable at: . Presently, MeDor provides a HCA plot and runs a secondary structure prediction, a prediction of signal peptides and transmembrane regions and a set of disorder predictions. MeDor also enables the user to customize the output and to retrieve the sequence of specific regions of interest.

**Conclusion:**

As MeDor outputs can be printed, saved, commented and modified further on, this offers a dynamic support for the analysis of protein sequences that is instrumental for delineating domains amenable to structural and functional studies.

## Background

In recent years there has been an increasing amount of experimental evidence pointing out the abundance of protein disorder within the living world. Recent computational studies have shown that the frequency and length of disordered regions increases with increasing organism complexity (see [1,2]), with as much as one third of eukaryotic proteins containing long intrinsically disordered regions [3] and 12% of them being fully disordered [4].

The identification of disordered regions has a practical interest. Disordered regions often have a biased amino acid composition that can lead to spurious sequence similarity with unrelated proteins. The recognition of regions of disorder is thus crucial to avoid spurious sequence alignments with sequences of unrelated, structured proteins. Secondly, the recognition of disordered regions facilitates the identification of eukaryotic linear motifs, which are short functional motifs occurring mainly (>70%) within disordered regions [5-8], and the functional annotation of proteins [9]. Last, but not least, disordered regions often prevent crystallization of proteins, or the generation of interpretable NMR data and thus represent a bottleneck in high throughput structural determination [10]. As such, their identification is instrumental for delineating protein domains amenable to crystallization and/or to dissect target sequences into a set of independently folded domains in order to facilitate tertiary structure and threading predictions (see [11,12]).

Intrinsically disordered proteins possess distinctive sequence features, including paucity of hydrophobic residues and enrichment in hydrophilic residues, which allow them to be predicted with a rather good accuracy. Based on these peculiar sequence properties, a series of predictors have been developed in the last years, the majority of which are available on the web (for reviews see [13-16]). As different "flavors" of disorder exist [17], a given predictor may be more performant in detecting a given "flavor" of disorder against which it has been trained. Hence, the reliability of disorder prediction benefits from the use of several methods relying on different concepts or different physico-chemical parameters. Indeed, we have previously shown that predictions good enough to decipher the modular organization of a protein can only be obtained by combining various predictors (for examples see [11,14,15,18-21]). However this is very time-consuming, since multiple predictions have to be carried out, individual outputs have to be retrieved, compared to each other and a comprehensive view of the results can only be obtained through a manual, fastidious, non-automated procedure. This prompted us to develop MeDor (MEtaserver of DisORder), which is a tool for speeding up the analysis of protein disorder thanks to the simultaneous submission and retrieval of multiple disorder predictions.

## Implementation

MeDor is a Java2 application that provides a graphical output summarizing the predictions of the following programs: a secondary structure prediction (SSP), based on the StrBioLib library of the Pred2ary program [22,23], Hydrophobic Cluster Analysis (HCA) [24], IUPred [25], Prelink [26], RONN [27], FoldUnfold [28], DisEMBL [29], FoldIndex [30], GlobPlot2 [31], DISPROT VL3 and VL3H [32], DISPROT VSL2B [33] and Phobius [34]. SSP and HCA have been included in the MeDor program and do not require a web connection. For predictors remotely launched through connection to the public web servers, we selected predictors that (i) rely on different physico-chemical principles, (ii) return results online with a delay compatible with the interactive character of the tool, and (ii) do not require an e-mail address. Additional predictors could be nevertheless easily implemented in MeDor in the future. Of the three predictions provided by DisEMBL, only Rem 465 provides disorder predictions [35] and is therefore run by default. The two other DisEMBL predictions, i.e. "Loops" and "Hot Loops", can be optionally selected from the MeDor input frame. These latter are indeed useful in terms of identification of regions devoid of regular secondary structure. As such, they are complementary to SSP, with the "Hot Loops" prediction providing in addition information on the extent of thermal agitation.

All requests are submitted in parallel by launching multiple predictors and using default parameters. Retrieval of results is fast, as it takes at maximum the time required by the slowest predictor to reply (in the limit of the timeout fixed by the user) *plus *the connection time to the server (limited to 15 seconds). SSP in MeDor is run using the medium jury of Pred2ary, which provides a good compromise between accuracy and rapidity. HCA makes use of a two-dimensional helical representation of protein sequences, and thus it is not *stricto sensu *a predictor. In HCA plots, disordered regions are recognizable as they are depleted in hydrophobic clusters [24].

For predictors that provide boundaries between ordered and disordered regions, these latter are directly extracted from the outputs. For predictors that provide only disorder probabilities, MeDor applies a 50% cutoff to assign disorder. Beyond disorder predictors, the Phobius program [34], which predicts transmembrane domains and signal peptides, can also be optionally selected from the MeDor input frame.

## Results

### Program input

The input format is a single protein amino acid sequence in either plain text or FASTA. The input window allows selection of the predictors to be run and choice of the timeout, which can be set in the range of 1 second to 30 minutes.

### Program output

**Figure 1:**
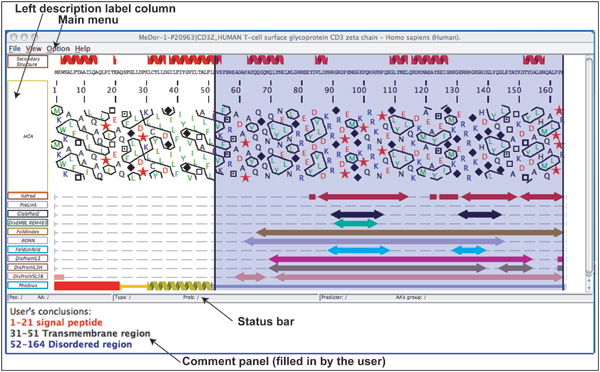
**Example of a MeDor output**. MeDor output of DisProt entry DP00200 human T cell glycoprotein CD3 Z chain (accession number P20963). The sequence is represented as a single, continuous horizontal line below the predicted secondary structure elements. Below the sequence are shown the HCA plot and the predicted regions of disorder that are represented by bidirectional arrows. Peptide signals and transmembrane domains predicted by Phobius are also highlighted as red bars and yellow helices, respectively. β-strands are represented by blue arrows, and α-helices are drawn in red. Predicted disordered regions are represented by bidirectional arrows of different colors as a function of predictors. The C-terminal region has been highlighted in blue. In the comment panel, the conclusions drawn by the user are shown as an example of comments that can be added.

MeDor provides a graphical output (Fig. 1), in which the sequence query and the results of the various predictors are featured horizontally, with a scroll bar allowing progression from the N-terminus to the C-terminus. All predictions are drawn along the sequence that is represented as a single, continuous horizontal line. Whenever provided by the disorder predictors, *per *residue probabilities are included in the MeDor output and shown in the status bar.

The graphical output is a dynamic interface that can be customized. It allows the user to display a graphical repository line for comparison among different predictions, to highlight zones of interest, to retrieve sequence boundaries for each prediction and to extract parts of sequences corresponding to a prediction or a highlighted area. The main menu of the graphical output gives access to several functions, such as focus in and out, display/hide the left description labels or the results of any of the predictors, select the highlight color, and display/hide the comment panel. This latter allows insertion of a text/comment in the rich text format (rtf). This main menu also contains options for printing the graphic output, for saving it either as a png image or as an XLM-based file format specific to MeDor (.med). The «Load» function from the input window menu of MeDor allows the user to load a file in the ".med" format. The whole MeDor functionalities are described in the associated help file.

## Conclusion

MeDor is not intended to provide a consensus of disorder prediction. Rather, it is conceived to provide a global overview of various predictions relying on different philosophies, and to speed up the disorder prediction step by itself. In addition to the fast identification of regions of disorder, MeDor can also be used to infer information on secondary structure elements and on the possible occurrence of transmembrane regions and signal peptides. Future developments of MeDor may involve generation of a consensus of disorder prediction, which is expected to further accelerate the process of deciphering the modular organization of proteins. Finally, as MeDor outputs can be saved, commented and modified further on, this offers a dynamic support for the analysis of protein sequences that is expected to be very useful in the context of collaborative projects involving several partners. As such, MeDor will facilitate the definition of domain boundaries amenable to structural and functional studies within proteins targeted by structural genomics consortia, such as VIZIER .

## Availability and requirements

Project name: MeDor

MeDor home page: 

Operating systems: Platform independent

Programming language: Java

Other requirements: Java 1.5.0 or higher and a web connection

License: This program uses predictions incoming from public web-servers and is provided freely and "as it is" without any warranty of any kind, either expressed or implied.

Any restrictions to use by non-academics: none.

## Competing interests

The authors declare that they have no competing interests.

## Authors' contributions

PL has designed, developed and implemented the MeDor program and participated to writing the manuscript and the program description material. BC provided advice and funding. SL had the original idea of developing a disorder metaserver and she wrote the manuscript and the description file.
